# General anesthetic management of a patient with multiple chemical sensitivity for oral surgery: a case report

**DOI:** 10.1186/s40981-019-0226-1

**Published:** 2019-02-14

**Authors:** Aiji Sato(Boku), Shota Furuno, Yuji Kamimura, Yoshiki Sento, Eisuke Kako, Masahiro Okuda, Yasuyuki Shibuya, Kazuya Sobue

**Affiliations:** 1 Department of Anesthesiology, Aichi Gakuin University School of Dentistry, 2-11 Suemori-dori Chikusaku, Nagoya, 464-8651 Japan; 2 Department of Oral and Maxillofacial Surgery, Nagoya City University Graduate School of Medical Sciences, 1 Kawasumi Mizuho-cho, Mizuho-ku, Nagoya, 467-8601 Japan; 3 Department of Anesthesiology and Intensive Care Medicine, Nagoya City University Graduate School of Medical Sciences, 1 Kawasumi Mizuho-cho, Mizuho-ku, Nagoya, 467-8601 Japan

**Keywords:** Multiple chemical sensitivity, Total intra venous anesthesia, Psychosomatic mechanisms

## Abstract

**Background:**

Multiple chemical sensitivity (MCS) was first described in 1987. It is said that MCS is caused by neurological and immunological mechanisms in addition to psychosomatic mechanisms. When performing general anesthesia in patients with MCS, careful perioperative management is necessary.

**Case presentation:**

The patient was a 32-year-old man. Wisdom teeth extraction under general anesthesia was scheduled under the diagnosis of pericoronitis. In 2015, he was diagnosed with MCS. Since then, he experienced sweating and urticaria when exposed to artificial fragrances. We prepared the surgical surroundings by letting the patient touch every possible equipment. In selecting the anesthetic drugs, a completely intravenous route was selected because of the possibility that artificial fragrance of inhalation anesthesia could induce symptoms. There was no allergic reaction during the preoperative period.

**Conclusions:**

It is important to reduce psychological burden of patient and to eliminate all possible reactive substances to prevent symptom onset.

## Background

Multiple chemical sensitivity (MCS) was first described by Cullen in 1987 and diagnostic criteria of MCS were defined **(**Table 1) [1]. There are no symptoms specific to the disease, and it is basically represented by subjective autonomic and psychoneurotic symptoms, such as malaise, fatigue, headache, arthralgia, insomnia, and dermatitis [2]. However, the mechanism of onset has not been clarified [3]. Hausteiner et al. hypothesized that MCS is a somatoform disorder [4]. In recent years, Kato suggested that MCS is caused by neurological and immunological mechanisms in addition to psychosomatic mechanisms [2]. At present, there are no treatment guidelines for MCS [5, 6]. When performing general anesthesia in patients with MCS, careful perioperative management is necessary because of the administration of multiple types of drugs. In this report, we describe our experience of performing general anesthesia in a patient with MCS for extraction of wisdom teeth. “Written consent” for this publication has been obtained from the patient.

**Table 1:** Seven major diagnostic features of the MCS by Cullen

1.The disorder is acquired in relation to some documentable environmental exposure(s), insult(s),or ileness(es).	
2. Symptoms involves more than one organ system.	
3. Symptoms recur and abate in response to predictable stimuli.	
4. Symptoms are elicited byexposures to chemicals of diverese structural classes and toxicological modes of action.	
5. Symptoms are elicited by exposures that are demonstrable (albeit of low level).	
6. Exposures that elicit symptoms must be very low, by which we mean many SDs below average exposures known to cause adverse human responses.	
7. No single widely available test of organ system function can explain the symptoms.	

## Case presentation

The patient was a 32-year-old man with a height of 167 cm and a weight of 52 kg. The patient complained of swelling and pain at the right lower third molar since September 2017. The patient was then referred to the Oral Surgery Clinic at Nagoya City University Hospital. Upon examination, due to the anatomical reasons, tooth extraction under general anesthesia was considered necessary although general anesthesia had a high risk of exposure to many chemical substances.

### Past medical history

**Fig. 1:**
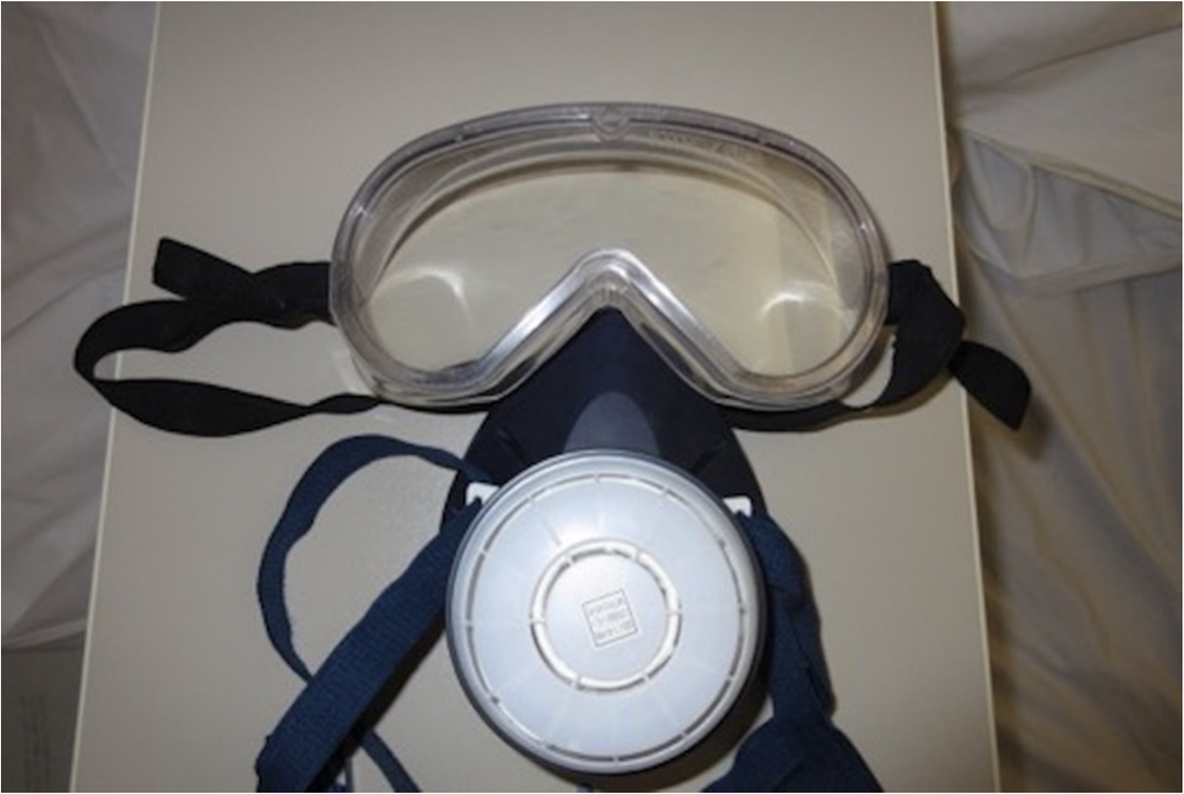
A sealed gas mask. The patient wears a sealed gas mask and rubber gloves in his daily life

The patient had repeated headaches, abnormal sweating, and loss of consciousness, which were considered to be caused by exposure to a coworker’s cigarette smoke from 2007 to 2013; he was diagnosed with recurrent acute passive smoking in 2014. In 2015, the patient had similar symptoms even after exposure to a smoke concentration below the reference value as determined by occupational administrative measures (suspended particles of 0.15 mg/m^3^) and was thus diagnosed with MCS. Since then, the patient experienced sweating and urticaria when exposed to artificial fragrances in shampoo or other substances and subsequently began wearing a sealed gas mask and rubber gloves in his daily life (Fig. 1). Table 2 shows the list of foods, chemicals, and other substances that the patient was intolerant to. The preoperative examination revealed no abnormal findings in chest radiography, electrocardiography, and blood tests. The patient was taking oral vitamins, bromazepam, and clonazepam.

**Table 2:** Lists the foods, chemicals, and other substances that the patient was intolerant to

1. Tea containing caffeine	
2. Juice containing fruit	
3. Soy sauce	
4. Chemical seasonings such as sauce and ketchup	
5. Wheat	
6. Chinese medecine	
7. Shampoo, rinse,softing agent contaning perfume	
8. Comercial mouthwash	

### Course of anesthesia

During preoperative evaluation before anesthesia, the patient reported sharing a space with a person who uses artificial fragrances such as detergent, fabric softener, or perfume, which causes him to sweat and develops urticaria. He indicated that his allergy to tobacco was the most severe, requiring him to completely avoid contact with smokers. In addition, any material that came in direct contact with the skin may have caused possible allergic reactions; however, this could not be determined until he actually came in contact with those materials. Based on the above history, our anesthetic goals included the following considerations:

1. Inhalation anesthetics would not be used because artificial fragrances were known to cause an allergic reaction.
2. To avoid contact with numerous unspecified individuals, the patient would be returned to the patient’s room upon awakening instead of being transferred to the post-anesthesia care unit (PACU).
3. Every piece of equipment to be used in surgery would be screened with the patient on the day prior to surgery, including artificial ventilation masks, sheets, towels, and tourniquets to ensure no allergic reaction would be caused.
4. Preparation with thorough measures such as adrenaline, antihistamine, and steroid against anaphylaxis.

**Fig. 2:**
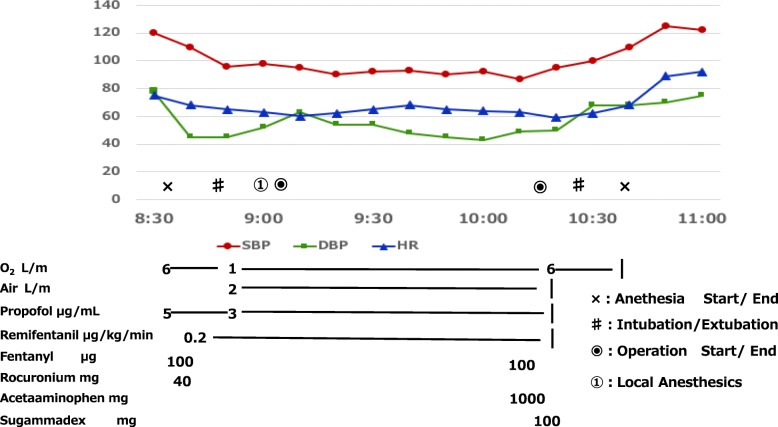
Anesthesia record. There was no allergic reaction during the preoperative period and there were no major issues

Because his allergic symptoms may have been induced by vaporized inhalation anesthetics from the previous day, his sealed gas mask was removed upon entering the operating room, and he was observed for 10 min. The patient did not exhibit any particular allergic symptoms, and after placing a standard monitor, a venous line was secured on the left forearm. Anesthesia was induced using propofol 5 μg/ml by target control infusion (TCI: Diprifusor model, TE-371, Terumo, Tokyo, Japan), fentanyl 100 μg, and remifentanil 0.2 μg/kg/min. After muscle relaxation was achieved using rocuronium 40 mg, nasal intubation was performed. We administered each drug after intervals of 3 min each. Anesthesia was maintained with oxygen, air, propofol 3 μg/ml (TCI), and remifentanil 0.2 μg/kg/min. As a local anesthetic, 2% lidocaine containing 1/80,000 diluted epinephrine was used. Hemodynamics was stable. Any hypotension, arrhythmias, respiratory problems, and neurological problems did not occur perioperatively. In addition, BIS, SpO2, and PETCO2 could adequately be controlled at 50–60, 100%, and around 40 mmHg, respectively, during anesthesia. Before the surgery was completed, 1000 mg of acetaminophen was administered. Following surgery, based on the value of TOF monitor, 100 mg of sugammadex was administered as a muscle relaxant antagonist, and the patient was extubated after confirming the patient’s response, eye opening, sufficient spontaneous breathing, and change in hemodynamics (Fig. 2). The total surgery lasted for 1 h 7 min, the anesthesia duration was 1 h 59 min, and the volume of blood loss was 5 ml. After surgery, the consciousness level and circulation dynamics were observed for 20 min in the operating room to confirm no abnormalities. The patient was returned to the general ward, bypassing the PACU. There was no allergic reaction during the preoperative period and there were no major issues.

## Discussion

In patients with MCS, various unexpected adverse events may make clinical evaluation difficult, thereby complicating therapeutic management [7]. There is little scientific documentation that indicates the safest protocol for anesthesia in patients with MCS [8], and to our knowledge, there are only four reports regarding general anesthesia in patients with MCS in recent years [7–10]. Thus, it is challenging for anesthesiologists to determine the appropriate drugs that do not cause or worsen symptoms. Therefore, the best approach is to prepare the surroundings and protect the patient from any substance that could be considered dangerous or cause inappropriate reactions [11, 12].

Several articles show that sevoflurane might be safety used for patient with MCS [7, 9].

However, our patient was sensitive to artificial fragrance. Therefore, we avoided all inhalation anesthesia because of the possibility that a fragrance could induce and worsen symptoms. On the other hand, Stoppe reported the xenon may be safe in patients with MCS [10]. Since xenon is odorless and nonirritating, it may have been effective for our patients, but it is not licensed in Japan and we cannot use it. For the reasons mentioned above, in selecting the anesthetic drugs, a completely intravenous route was selected. Furthermore, in the present case, we prepared the surgical surroundings by letting the patient touch every possible equipment item the day before surgery and monitored possible reactions. In addition, as the patient had a known sensitivity to tobacco, staff who smoked tobacco were not permitted to enter the operating room. Moreover, hair styling products and fragrances were banned for staff on the day of surgery.

In addition to preparing the surroundings, we brought the patient into the operating room wearing a gas mask. After entering the operating room, the gas mask was removed, and the patient was exposed to the air in the operating room for approximately 10 min; he exhibited no adverse reaction. These efforts to prepare the surgical and recovery surroundings was undertaken to prevent the onset of symptoms.

Psychosomatic mechanisms have been suggested as an onset mechanism of MCS. In fact, the patient was routinely taking antianxiety drugs; thus, it was necessary to minimize anxiety as much as possible before surgery. We let the patient touch as many pieces of equipment as possible to reduce his psychological stress. In addition, loss of consciousness due to general anesthesia also contributed to the prevention of attacks.

## Conclusions

We described our experience of general anesthesia in a patient with MCS for wisdom tooth extraction. It is important to eliminate all possible reactive substances from the patient’s room to the operating room to prevent symptom onset and reduce psychological burden of patients.

## Abbreviations

MCS: Multiple chemical sensitivity
PACU: Post anesthesia care unit
TCI: Target control infusion
